# Oncologic outcomes after immediate breast reconstruction following mastectomy: comparison of implant and flap using propensity score matching

**DOI:** 10.1186/s12885-020-6568-2

**Published:** 2020-01-30

**Authors:** Jeong Hyun Ha, Ki Yong Hong, Han-Byoel Lee, Hyeong-Gon Moon, Wonshik Han, Dong-Young Noh, Joonho Lim, Sehoon Yoon, Hak Chang, Ung Sik Jin

**Affiliations:** 1 Department of Plastic and Reconstructive Surgery, Seoul National University Hospital, Seoul, South Korea; 2 Department of Plastic and Reconstructive Surgery, Dongguk University Medical Center, Goyang, South Korea; 3 Department of Surgery, Seoul National University College of Medicine, Seoul, South Korea; 4 Cancer Research Institute, Seoul National University, Seoul, South Korea; 5 Department of Plastic and Reconstructive Surgery, Seoul National University College of Medicine, Seoul, South Korea

**Keywords:** Breast neoplasm, Breast reconstruction, Flap, Implant, Oncologic safety

## Abstract

**Background:**

Although immediate breast reconstruction has been reported to be oncologically safe, no affirmative study comparing the two reconstruction methods exists. We investigated breast cancer recurrence rates in two breast reconstruction types; implant reconstruction and autologous flap reconstruction.

**Methods:**

A retrospective cohort study was performed on propensity score-matched (for age, stage, estrogen receptor status) patients who underwent IBR after mastectomy at Seoul National University Hospital between 2010 and 2014. The main outcomes determined were locoregional recurrence-free interval (LRRFI) and disease-free interval (DFI).

**Results:**

We analyzed 496 patients among 731 patients following propensity score matching (Median age 43, 247 implant reconstruction and 249 flap reconstruction). During median follow-up of 58.2 months, DFI was not different between the two groups at each tumor stage. However, flap reconstruction showed inferior DFI compared to implant reconstruction in patients with high histologic grade (*p* = 0.012), and with high Ki-67 (*p* = 0.028). Flap reconstruction was related to short DFI in multivariate analysis in aggressive tumor subsets. Short DFI after flap reconstruction in aggressive tumor cell phenotype was most evident in hormone positive/Her-2 negative cancer (*p* = 0.008). LRRFI, on the other hand, did not show difference according to reconstruction method regardless of tumor cell aggressiveness.

**Conclusion:**

Although there is no difference in cancer recurrence according to reconstruction method in general, flap-based reconstruction showed higher systemic recurrence associated with histologically aggressive tumors.

## Background

A combination of surgical excision, systemic chemotherapy, and radiation therapy are applied in breast cancer to improve the oncologic outcome. Immediate breast reconstruction (IBR) has become the mainstay method of aesthetic and functional improvement following mastectomy for breast cancer [1]. It is essential that breast reconstruction be safe from an oncologic point of view. In other words, IBR should not increase the risk of relapse or hinder subsequent anti-cancer treatment such as adjuvant chemotherapy by causing complications [2]. Previous studies have tried to establish the oncologic safety of IBR, showing that IBR did not increase recurrence rates or delay the detection of recurrence [3, 4]. However, they did not differentiate the outcomes according to specific IBR methods or tumor histologies [3, 5, 6]. To our knowledge, no one has yet performed a matched case-control study comparing locoregional and distant metastasis rates between reconstruction methods.

It is well established that breast reconstruction provides psychological benefits and improvements in quality of life [7]. Nevertheless, IBR has traditionally not been recommended over delayed reconstruction [8]. The reluctance to use IBR originates from concerns that IBR might increase the risk of locoregional recurrence and that recurrence might be more difficult to detect after IBR [6]. However, recent reports suggest that IBR is oncologically safe in invasive breast cancer and is practiced widely [3, 4, 9–11]. Although the locoregional recurrence rates after IBR vary across studies, it is commonly accepted that IBR does not significantly increase recurrence. For example, an anecdotal study by Eriksen et al. [6] reported that implant-based IBR did not affect oncologic outcomes including locoregional and distant recurrence. The authors compared locoregional and distant recurrence between patients that underwent implant-based IBR and a mastectomy-only group in this study. Likewise, Howard et al. [3] analyzed oncologic outcomes between patients that underwent TRAM flap-based IBR and a mastectomy-only control group. In the study, there was no difference in the local recurrence rate between the two groups.

Implant-based breast reconstruction is a safe method with favorable outcomes, minimal morbidity, and short operative times. On the other hand, flap-based breast reconstruction is performed using microvascular free flap transfer or pedicled flap transfer. Reports suggest that flap reconstruction has certain advantages over implant-based reconstruction, such as a lower complication rate during adjuvant radiotherapy and better long-term aesthetic outcomes [12–15]. Some surgeons prefer flap reconstruction over implant-based reconstruction when the cancer is in a locally advanced stage, because the former is less likely than the latter to cause complications during adjuvant therapy [16]. In general, patient-specific factors such as breast size, degree of ptosis, comorbidity, age, and patient preference are used to determine the best method of reconstruction for each patient [17].

Although recent reports suggest that IBR does not affect cancer recurrence or detection [3, 5, 18] there are still oncologic concerns about the use of flap reconstruction [19]. There are no affirmative data comparing locoregional recurrence rates between implant-based reconstruction and flap reconstruction. Although neither method is reported to increase the relapse risk in general, the oncologic safety of flap reconstruction still needs to be more precisely analyzed from diverse perspectives. The aim of this study was to investigate whether there is a difference in locoregional and overall recurrence between those two reconstruction methods in patients with breast cancer who undergo IBR.

## Methods

### Patients

We identified all patients who underwent IBR at Seoul National University Hospital (SNUH) from 2010 to 2014. We reviewed the patients’ demographics and oncologic and reconstructive data after receiving approval from the Institutional Review Board of Seoul National University Hospital (IRB No. H-1602-132-744). We excluded patients with pathologic results indicating phyllodes tumor, angiosarcoma, or metastatic cancer at initial presentation; those who underwent prophylactic mastectomy; and those with prior history of breast cancer. We excluded patients with major complications such as flap loss or implant loss that may delay adequate postoperative anti-cancer treatment to achieve pure oncologic comparisons of the two reconstruction methods. In other words, we assumed that all implant or flap reconstructions were properly performed, assessing oncologic effects of each reconstruction method in terms of hemodynamic or immunologic flux, rather than focusing on pragmatic outcomes. Cases converted to flap reconstruction after tissue expander insertion were also excluded. The primary endpoint in our study was locoregional recurrence or distant metastasis.

### Comparison of clinical outcomes using propensity-score matching

We grouped the patients into two cohorts according to IBR method: (1) patients who underwent IBR with implant (including tissue expander), and (2) patients who underwent IBR with flap transfer. For the comparison of oncologic outcomes between the two IBR methods, we conducted propensity score matching. We calculated the propensity scores by logistic regression analysis including age, American Joint Committee on Cancer (AJCC) 7th ed. tumor staging [20], and estrogen receptor (ER) status. We matched patients by propensity score using the nearest-neighbor method with a matching ratio of 1:1. The caliper width was equal to 0.2 times the standard deviation of the logit of the propensity score. After matching, we reviewed the covariate balance for statistical significance and standardized difference.

### Immunohistochemistry

ER, progesterone receptor (PR), human epidermal growth factor receptor-2 (HER2), histologic grade (HG), nuclear grade (NG), and Ki-67 expressions were evaluated. ER, PR and HER2 was evaluated following ASCO/CAP Guideline [21–23]. HG was graded according to Nottingham classification [24]. The percentage of Ki-67 was determined by the number of Ki-67 positive cells among the total number of counted tumor cells. High expression of Ki-67 was defined as ≥10%, based on the previous study in our institution [25, 26].

### Operative technique

We performed IBR using implant or flap after mastectomy. In the implant-based reconstructions, we inserted an implant (or tissue expander) according to the amount of skin resected during the mastectomy. We inserted the implant at the submuscular layer and used an acellular dermal matrix (ADM) [CG CryoDerm (CGBio Corp., Seongnam, Korea) or DermACELL (LifeNet Health, Virginia Beach, VA, USA)] to cover the inferolateral aspect. In the flap reconstructions, we transferred either a free vascularized flap or a pedicled flap (e.g., from the rectus abdominis or latissimus dorsi myocutaneous flap).

### Statistical analysis

Statistical analyses of 2 × 2 contingency tables of categorical variables were performed as appropriate using Fisher’s exact test or Pearson’s χ^2^ test. We calculated the mean durations of survival using the Kaplan-Meier method. Comparisons between groups were performed using log-rank tests. We used logistic regression and a Cox regression model to analyze the effects of continuous numeric variables on clinical outcomes. Multivariate analysis was achieved using logistic regression and Cox regression with factors that showed *p*-values < 0.1 in the univariate analyses. Locoregional relapse-free interval (LRRFI) was defined as the time between the breast cancer surgery and the detection of locoregional recurrence by biopsy or imaging. Disease-free interval (DFI) was defined as the time between breast cancer surgery and the detection of any relapse. We focused on DFI rather than DFS because there were a few deaths not related to breast cancer which would askew oncologic outcome in this subset. All statistical tests were two-sided with *p* < 0.05 as the threshold for statistical significance. Analyses were performed using the Statistical Package for the Social Sciences for Windows Version 21.0 (IBM, Chicago, IL, USA).

## Results

### Baseline characteristics and propensity score matching

Between January 2010 and December 2014, 731 patients underwent IBR after mastectomy at SNUH for primary breast cancer. A total of 664 patients who meet study criteria underwent propensity score matching based on age, cancer stage [20], and ER status, which resulted in the inclusion of 496 patients (247 implants and 249 flaps) for further analysis. (Additional file 1: Fig. S1).

There were no differences between the two groups in cancer stage, weight of excised breast mass, chemotherapy or radiotherapy status, axillary lymph node status, ER status, PR status, NG, HG, and HER2 amplification after propensity score matching (Table 1*,* Additional file 2: Table S1). Of the 247 patients from implant group, 60 (24.3%) patients received implant insertion, and 187 (75.7%) received tissue expander insertion. The majority of flap reconstructions used a free transverse rectus abdominis myocutaneous (TRAM) flap (*n* = 238, 95.6%); other used a pedicled latissimus dorsi myocoutaneous flap (*n* = 7, 2.8%), a free superficial inferior epigastric artery perforator flap (*n* = 1, 0.4%), a free inferior gluteal artery perforator flap (n = 1, 0.4%), a free gracilis flap (*n* = 1, 0.4%), or a free lumbar artery perforator flap (n = 1, 0.4%).

**Table 1:** Patients demographics

Characteristics		Reconstruction type		*p*-value
		Implant* (*n* = 247)	Flap (*n* = 249)	
Age, years				0.190
	Average	41 ± 8.73	43 ± 6.99	
Diagnosis				0.076
	IDC	164	172	
	DCIS	46	50	
	ILD	17	8	
	LCIS	0	4	
	Mixed	11	8	
	Others	9	7	
Mastectomy type				0.151
	TM	115	107	
	SSM	64	84	
	NSM	68	58	
Excised breast mass, gram				0.264
	Average	367 ± 191.63	409 ± 510.13	
Lymph node status				0.782
	N0	180	171	
	N1	43	51	
	N2	16	18	
	N3	8	9	
AJCC Stage				0.278
	0	47	57	
	IA	99	82	
	IB	1	0	
	IIA	49	53	
	IIB	24	26	
	IIIA	20	19	
	IIIB	0	3	
	IIIC	7	9	
Chemotherapy				0.647
	none	111	117	
	perioperative	136	132	
Radiotherapy				0.702
	none	195	200	
	adjuvant	51	48	
Nuclear grade, no. (%)				0.487
	1	4	4	
	2	101	84	
	3	94	100	
Histological grade, no. (%)				0.896
	1	16	14	
	2	104	95	
	3	74	74	
ER, no. (%)				0.462
	Negative	49	43	
	Positive	198	206	
PR, no. (%)				0.952
	Negative	76	76	
	Positive	171	173	
HER2, no. (%)				0.128
	Negative	174	193	
	Positive	56	44	
Ki67, no. (%)				0.014
	< 10%	198	174	
	≥10%	48	71	

*IDC* invasive ductal carcinoma; *ILC* invasive lobular carcinoma; *DCIS* ductal carcinoma in situ; *LCIS* lobular carcinoma in situ; *TM* total mastectomy; *SSM* skin-sparing mastectomy; *NSM* nipple-sparing mastectomy; *AJCC* American Joint Committee on Cancer; *ER* estrogen receptor; *PR* progesterone receptor; *HER2* human epidermal growth factor receptor-2

*Implant group includes patients who received reconstruction with tissue expander

### Cancer recurrence after reconstruction

During median follow-up duration was 58.2 months (57.3 and 58.3 months for implant and flap group, respectively) there were 38 recurrence events. Cancer stage was an independent prognostic factor for recurrence (*p* < 0.001). The NG (*p* = 0.004), HG (*p* = 0.001), and Ki-67 (*p* < 0.001) were also prognostic factors for cancer recurrence. Vascular emboli and lymphatic emboli affected the DFI (*p* < 0.001 and *p* < 0.001, respectively); however, ER status (*p* = 0.172), PR status (*p* = 0.190), and HER-2 status (*p* = 0.642) did not.

**Fig. 1:**
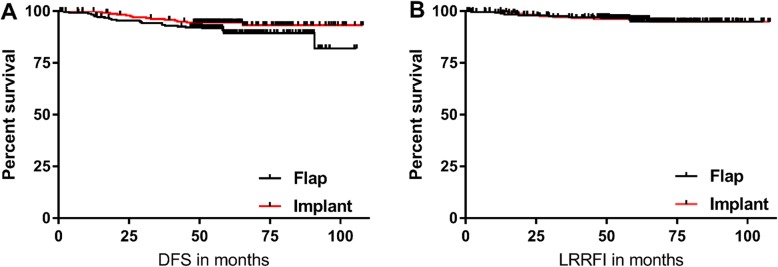
Disease free survival (**a**) and Locoregional relapse free interval (**b**) in implant and flap based immediate breast reconstruction

There was no difference in the DFI between implant group and flap group. During follow up, 14 patients relapsed in implant group and 24 patients relapsed in flap group. The 5-year DFI rate was 93% in implant group and 90% in flap group (*p* = 0.100) (Fig. 1a). There were no differences in DFI between the patients that underwent one-stage implant and two-stage expander insertion (*p* = 0.861) or between those that underwent TRAM flap and other types of flap reconstructions (*p* = 0.859).

In a multivariate analysis for DFI including cancer stage, NG, HG, and Ki-67, cancer stage (*p* = 0.007) was an independent prognostic factor (Additional file 3: Table S2).

### Systemic cancer recurrence affected by IBR method in aggressive tumors

**Fig. 2:**
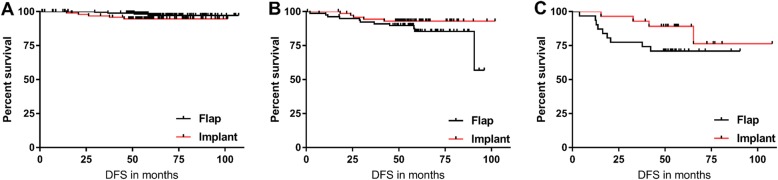
Disease free survival in (**a**) Stage I, (**b**) Stage II, and (**c**) Stage III patients according to breast reconstruction type

When we considered the different cancer stages separately, there was no difference in DFI between the implant and flap group (*p*-value for stage 1 = 0.642; stage 2 = 0.195; stage 3 = 0.132) (Fig. 2).

**Fig. 3:**
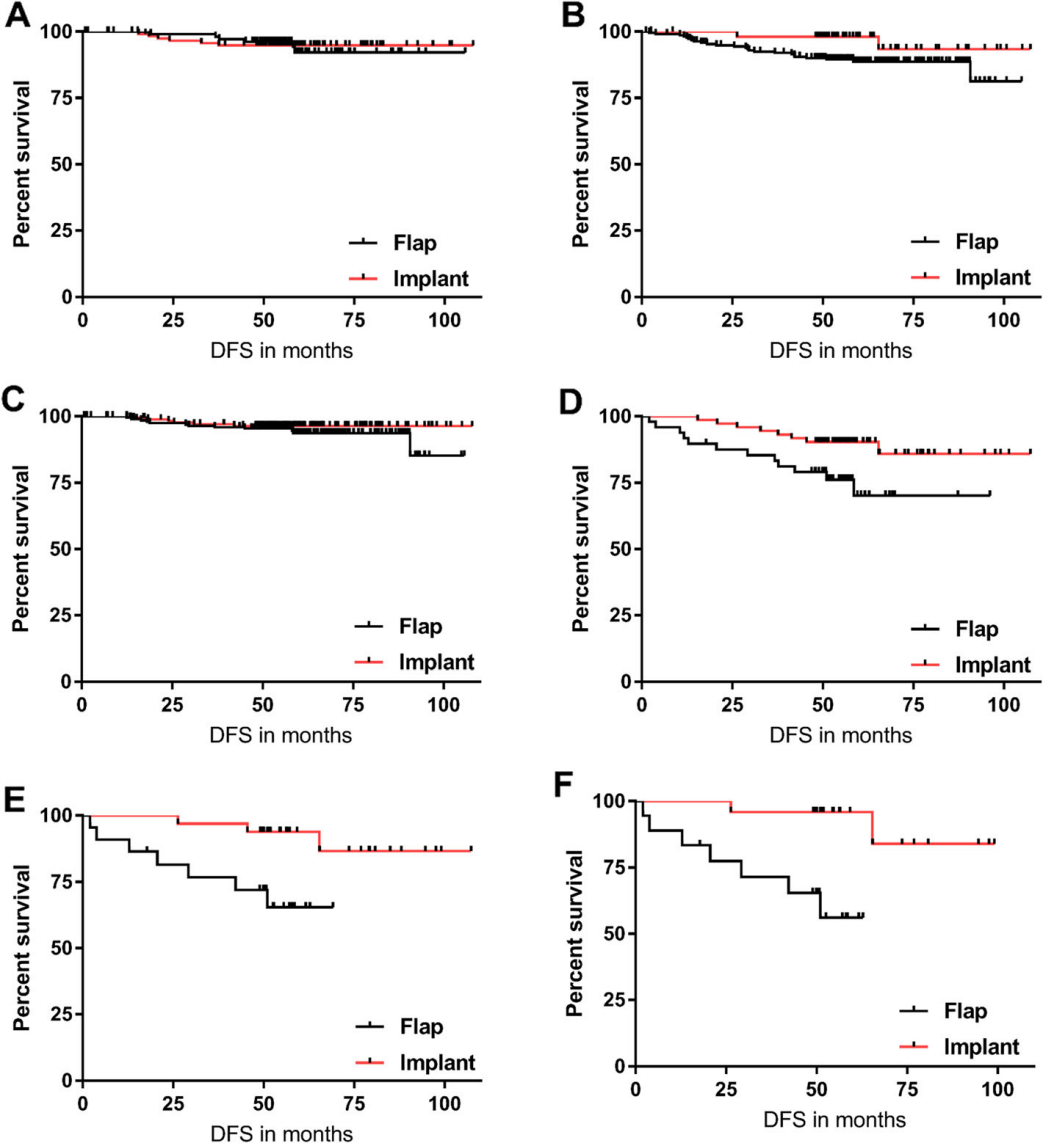
Disease free interval (DFI) in (**a**) Histologic grade 1–2, (**b**) Histologic grade 3, (**c**) Ki-67 < 10%, (**d**) Ki-67 ≥ 10% patients according to breast reconstruction type. DFI of aggressive tumor (high histologic grade and high Ki-67) in (**e**) hormone positive, (**f**) hormone positive/Her2-negative patients according to breast reconstruction type

On the other hand, when we considered the HG separately, patients with HG 3 (high HG) in flap group (*n* = 74) had a lower 5-year DFI rate than implant group (*n* = 75) (5-year DFI rate for implant group 92% vs. flap group 77%; *p* = 0.012). There was no such difference among the patients with HG 1 or 2, however (*p* = 0.917). Likewise, flap reconstruction showed short DFI in patients with high Ki-67 (*p* = 0.028). In contrast, there was no difference in DFI between the two groups in low Ki-67 (*p* = 0.278). When both HG and Ki-67 were considered, aggressive tumor (defined by high HG and high Ki-67) relapsed more frequently following flap reconstruction than implant reconstruction (*p* = 0.004) (Fig. 3a-d). Patient characteristics between the two reconstruction groups in high HG and/or high Ki-67 group did not differ.

In multivariate analysis for DFI performed within high HG group considering cancer stage, hormone receptor (HR), HER2 and reconstruction type, the reconstruction type was the independent prognostic factor (*p* = 0.018) (Table 2). Likewise, in high Ki-67 group, the reconstruction type was the independent prognostic factor for DFI in multivariate analysis (*p* = 0.015, data not shown).

**Table 2:** Hazard ratio and p-value of disease-free interval in high histologic grade (grade 3) group using a Cox proportional hazard model in multivariate analysis

Characteristics	HR	95% CI		
		Lower	Upper	*p*-value
Reconstruction type				0.018
Prosthesis	1.00			
Flap	3.39	1.23	9.32	
Stage				0.040
I	1.00			
II	1.79	0.56	5.78	
III	4.55	1.32	15.67	
Hormone receptor				0.491
Positive	1.00			
Negative	1.42	0.52	9.32	
HER2				0.430
Positive	1.52	0.54	4.33	
Negative	1.00			

*HR* Hazard ratio; *CI* confidence interval; *HER2* human epidermal growth factor receptor-2

When HR and HER2 status was considered, DFI was not different between two groups in each tumor subtype: including triple-negative breast cancer (TNBC; *p* = 0.668), and HR-positive breast cancer (*p* = 0.230). However, in 71 aggressive tumors (high HG and high Ki-67), frequent relapse after flap reconstruction was seen especially in HR-positive breast cancers (HR-positive: *p* = 0.008; HR-positive/HER2-negative: *p* = 0.002), which accounts for majority of our study population (Fig. 3e-f).

Next, we observed whether the reconstruction type affected locoregional recurrence. There were 20 locoregional recurrences during follow up: 9 in implant and 11 in flap group. The 5-year LRRFI rate was 95% in implant group and 95% in flap group (*p* = 0.991) (Fig. 1b). Unlike the DFI, the LRRFI was not affected by reconstruction method neither in high HG tumor (*p* = 0.445) nor in high Ki-67 tumor (*p* = 0.791). The reconstruction type did not affect locoregional recurrence in a multivariate analysis (*p* = 0.704).

## Discussion

IBR is widely performed and previous studies have tried to establish the oncologic safety of IBR, showing that IBR did not increase recurrence rates or delay the detection of recurrence [3, 4]. However, those previous reports did not differentiate the outcomes according to specific IBR methods or tumor histologies [3, 5, 6]. To our knowledge, no one has yet performed a matched case-control study comparing locoregional and distant metastasis rates between reconstruction methods. There may be, however, a chance of tumor spreading through vascular anastomosis of flap with surrounding breast envelope, raising doubts about oncologic safety. The hemodynamic environment, which may influence tumor spreading, is presumed to be different between the two reconstruction methods. Hence, although neither method is reported to increase the relapse risk in general, the oncologic safety of flap reconstruction still needs to be more precisely analyzed from diverse perspectives.

In this regard, we performed a propensity-matched, case-control study to compare oncologic safety between implant-based and flap reconstructions: we focused on DFI rather than DFS because there were a few deaths not related to breast cancer which would askew oncologic outcome in this subset. We excluded patients with major complications that may delay adequate postoperative anti-cancer treatment to achieve pure oncologic comparisons of the two reconstruction methods. In other words, we assumed that all implant or flap reconstructions were properly performed, assessing oncologic effects of each reconstruction method in terms of hemodynamic or immunologic flux, rather than focusing on pragmatic outcomes. We did not find any difference in DFI between the two reconstruction methods. We can therefore conclude that in general, the choice of reconstruction method does not affect oncological outcome. In addition, there were some unexpected findings in our subgroup analyses.

Among patients with high HG, the DFI was shorter in the flap group than in the implant group. In line with histologic grading, patients with high Ki-67 showed shorter tendency for DFI in flap group compared to implant group. This was confirmed both in univariate and multivariate analysis. When we combined HG and Ki-67 to define tumor aggressiveness, aggressive tumor showed higher rate of relapse following flap reconstruction than implant-based reconstruction. On the other hand, locoregional recurrence was not different based on reconstruction method among patients with aggressive histology suggesting that flap reconstruction was related to the systemic recurrence. There was no difference in chemotherapy status or cancer stage between the two reconstruction groups with high HG tumor (*n* = 148), which excludes the possibility of selection bias in that finding. Due to small number of absolute relapse events, affirmative conclusion could not be drawn with this single study. However, it should be noted from this study that high HG tumor may have increased rate of systemic relapse with flap reconstruction which accompanies increased vascularity around surgical bed. Surgical stress imposed by flap operations may thus foster distant relapse of aggressive tumors, as in a mouse model of breast cancer [27]. Because perioperative immunomodulation, stemming from surgical stress, may figure prominently in antimetastatic immune activity [28, 29], it is not surprising that flap reconstruction, which entails prolonged operative time and therefore more surgical stress than implant reconstruction, is associated with shorter DFIs. Distant recurrences may be cultivated from preexisting micrometastases in aggressive cancer cell types due to perioperative immunosuppression [28, 29]. In fact, HG [30] and Ki-67 [31] are well-known prognostic factors for breast cancer. But we could not find similar findings as ours in the literature and concluded that it was because previous research did not focus on HG or Ki-67. Eriksen et al. [6], Howard et al. [3], and McCarthy et al. [5] all analyzed the oncologic outcomes of implant or flap-based IBR. However, data regarding the HG and Ki-67 were not assessed in all studies. It should be noted that, on the other hand, DFI was not different depending on the reconstruction method when the analysis was stratified on other tumor characteristics such as cancer stage, nuclear emboli, or TNBC. This suggests that the tumor aggressiveness at the single-cell level represented by high HG or high Ki-67 is the most important factor in the increased risk of relapse after flap reconstruction in breast cancer.

We tried to overcome limitations of our study design. First, there was a possibility of bias in the patient characteristics between the two groups. Young patients may more likely want to undergo implant-based reconstruction for simultaneous augmentation of contralateral breast. On the other hand, surgeons might prefer flap reconstruction in advanced cancer, considering the possible radiation-related complications associated with implants. We tried to minimize this bias by using propensity score matching for more balanced comparison. Second, majority of our study population were HR-positive and HER2-negative. We could observe short DFI after flap reconstruction in aggressive tumor cell phenotype within this subgroup but not in other subgroups with statistical significance. Hence, effect on recurrence according to reconstruction method in less representative subtypes in our study should be analyzed in further studies. Lastly, because this was a retrospective study, we cannot be certain that some information that might have influenced the outcome was not left out. One example is tumor size, but data concerning tumor size or the ratio of tumor size to breast size were not available for analyses. In addition, how the reconstruction method was chosen is another important factor that could not be assessed because of the retrospective nature of the study. However, as several previous important retrospective studies [32, 33], we assume discovering novel findings from retrospective cohort study is virtually not impossible. We hope further study with robust research design (e.g. prospective study) would consolidate our anecdotal findings.

## Conclusions

We report that there is no difference in cancer recurrence according to the method used for immediate breast reconstruction in general. There is, however, a possibility that flap reconstruction increases the risk of systemic recurrence in high HG and/or high Ki-67 tumors. Our study suggests that when breast cancers is revealed to have high HG or high Ki-67 upon preoperative biopsy, flap reconstruction after mastectomy should be performed with caution from an oncologic point of view.

## Supplementary information

**Additional file 1.** **Fig. S1**. CONSORT flow diagram

**Additional file 2:** **Table S1.** Patient demographics before propensity score matching

**Additional file3: Table S2.** Hazard ratio and *p*-value of disease-free interval using a Cox proportional hazard model in multivariate analysis

## Data Availability

All data generated or analyzed during this study are included in this published article and its supplementary information files.

## Abbreviations

AJCC: American Joint Committee on Cancer, 7th ed
DFS: Disease-free survival
ER: Estrogen receptor
HER2: Human epidermal growth factor receptor 2
HG: Histologic grade
HR: Hormone receptor
IBR: Immediate breast reconstruction
LRRFI: Locoregional recurrence-free interval
NAC: Nipple-areola complex
NG: Nuclear grade
NSM: Nipple-sparing mastectomy
PR: Progesterone receptor
SSM: Skin-sparing mastectomy
TM: Total mastectomy
TRAM: Transverse rectus abdominis myocutaneous
