# Supplementary material for: Oncologic outcomes after immediate breast reconstruction following mastectomy: comparison of implant and flap using propensity score matching
Source: BMC Cancer. 2020 Jan 30;20:78. doi: 10.1186/s12885-020-6568-2 (PMC6993337; doi:10.1186/s12885-020-6568-2)
Supplement: Supplementary file 2 — Additional file 2: Table S1. Patient demographics before propensity score matching [file 12885_2020_6568_MOESM2_ESM.docx]

Supplementary Table 1. Patient demographics before propensity score matching

| **Characteristics** | | **Reconstruction type** | | **Total** | **p-value** |
| --- | --- | --- | --- | --- | --- |
|  |  | **Implant* (n=255)** | **Flap (n=409)** |  |  |
| **Age** | Average | 41 | 45 |  |  |
|  | SD | 8.68 | 7.15 |  |  |
| **AJCC Stage** |  |  |  |  | 0.024 |
|  | 0 | 53 | 79 | 132 |  |
|  | I | 100 | 118 | 218 |  |
|  | II | 74 | 150 | 224 |  |
|  | III | 29 | 61 | 90 |  |
| **Nuclear grade** |  |  |  |  | 0.099 |
|  | 1 | 4 | 8 | 12 |  |
|  | 2 | 102 | 132 | 234 |  |
|  | 3 | 95 | 181 | 276 |  |
| **Histologic grade** |  |  |  |  | 0.191 |
|  | 1 | 17 | 20 | 37 |  |
|  | 2 | 104 | 144 | 248 |  |
|  | 3 | 75 | 141 | 216 |  |
| **ER status** |  |  |  |  | 0.01 |
|  | Negative | 49 | 108 | 157 |  |
|  | Positive | 200 | 267 | 467 |  |
| **PR status** |  |  |  |  | 0.031 |
|  | Negative | 77 | 147 | 224 |  |
|  | Positive | 172 | 226 | 398 |  |
| **HER2 status** |  |  |  |  |  |
|  | Negative | 174 | 294 | 468 |  |
|  | Positive | 57 | 96 | 153 |  |

AJCC, American Joint Committee on Cancer; ER, estrogen receptor; PR, progesterone receptor; HER2, human epidermal growth factor receptor-2

*Implant group includes patients who received reconstruction with tissue expander
