# Supplementary material for: Oncologic outcomes after immediate breast reconstruction following mastectomy: comparison of implant and flap using propensity score matching
Source: BMC Cancer. 2020 Jan 30;20:78. doi: 10.1186/s12885-020-6568-2 (PMC6993337; doi:10.1186/s12885-020-6568-2)
Supplement: Supplementary file 3 — Additional file3: Table S2. Hazard ratio and p-value of disease-free interval using a Cox proportional hazard model in multivariate analysis [file 12885_2020_6568_MOESM3_ESM.docx]

Supplement Table 2. Hazard ratio and p-value of disease-free interval using a Cox proportional hazard model in multivariate analysis

| **Characteristics** | **HR** | **95% CI** | | |
| --- | --- | --- | --- | --- |
|  |  | **Lower** | **Upper** | **p-value** |
| Stage |  |  |  | 0.006 |
| I | 1.00 |  |  |  |
| II | 2.47 | 0.96 | 6.36 |  |
| III | 5.38 | 1.91 | 15.18 |  |
| Hormone receptor |  |  |  | 0.668 |
| Positive | 0.83 | 0.34 | 1.98 |  |
| Negative | 1.00 |  |  |  |
| HER2 |  |  |  | 0.839 |
| Positive | 1.00 |  |  |  |
| Negative | 0.83 | 0.41 | 2.08 |  |
| Ki-67 |  |  |  | 0.469 |
| High | 1.34 | 0.61 | 2.92 |  |
| Low | 1.00 |  |  |  |
| Nuclear grade |  |  |  | 0.326 |
| 1,2 | 1.00 |  |  |  |
| 3 | 1.80 | 0.56 | 5.78 |  |
| Histologic grade |  |  |  | 0.503 |
| 1,2 | 1.00 |  |  |  |
| 3 | 1.46 | 0.48 | 4.44 |  |

HR, Hazard ratio; CI, confidence interval; HER2, human epidermal growth factor receptor-2
